# The Design of Sulfated Ce/HZSM-5 for Catalytic Decomposition of CF_4_

**DOI:** 10.3390/polym14132717

**Published:** 2022-07-02

**Authors:** Xie Zheng, Shijie Chen, Wanning Liu, Kaisong Xiang, Hui Liu

**Affiliations:** 1 School of Metallurgy and Environment, Central South University, Changsha 410083, China; zhengxie@csu.edu.cn (X.Z.); csj19940415@163.com (S.C.); liuwanning@csu.edu.cn (W.L.); leolau@csu.edu.cn (H.L.); 2 Chinese National Engineering Research Center for Control & Treatment of Heavy Metal Pollution, Changsha 410083, China; 3 School of Chemistry and Chemical Engineering, Central South University, Changsha 410083, China

**Keywords:** CF_4_, perfluorocompounds, catalytic decomposition, Lewis acid site, cerium, sulfuric acid treatment

## Abstract

CF_4_ has a global warming potential of 6500 and possesses a lifetime of 50,000 years. In this study, we modified the HZSM-5 catalyst with Ce and sulfuric acid treatment. The S/Ce/HZSM-5 catalyst achieves 41% of CF_4_ conversion at 500 °C, which is four times higher than that over Ce/HZSM-5, while the HZSM-5 exhibits no catalytic activity. The effects of modification were studied by using NH_3_-TPD, FT-IR of pyridine adsorption, and XPS methods. The results indicated that the modification, especially the sulfuric acid treatment, strongly increased the Lewis acidic sites, strong acidic sites, and moderate acidic sites on catalysts, which are the main active centers for CF_4_ decomposition. The mechanism of acidic sites increases by modification and CF_4_ decomposition is clarified. The results of this work will help the development of more effective catalysts for CF_4_ decomposition.

## 1. Introduction

Perfluorocarbon (PFC) is a class of greenhouse-gas (GHG) in which all valences of carbon are satisfied with fluorine atoms [1,2]. CF_4_ is considered as the most abundant and harmful in PFCs, and it has a high global warming potential, which is about 6500 times higher than that of CO_2_ over a 100-year time scale [3]. Due to its symmetry and strong ionic character in the C–F covalent bond, which is the strongest bond (543 kJ mol^−1^) in organic chemistry, the CF_4_ molecule is extremely stable [4]. The aluminum production industry is considered as the main source of CF_4_ emission. When the anode effect occurs in the electrolytic cell, the flux Na_3_AlF_6_ will react with the C anode to form CF_4_ as in the equation below [5]. In 2018, the global CF_4_ emission from aluminum production is 4.408 Gg, accounting for approximately 4% of total GHG emission in CO_2_ equivalent [6]. However, the lifetime of CF_4_ in atmosphere is 50,000 years while that of CO_2_ is only 10–20 years. Therefore, it is necessary and important to remove CF_4_ presented in the exhaust flue gas from the aluminum production industry.

4Na_3_AlF_6_ + 3C → 4Al + 12NaF + 3CF_4_

To date, several methods have been developed for CF_4_ abatement, such as fueled combustion, plasma, and catalytic hydrolytic decomposition [7,8,9]. Among them, the catalytic hydrolytic decomposition is considered as the ideal method for its high efficiency and mild reaction temperature compared to others.

HZSM-5 is a molecular sieve catalyst with Si, Al, and O elements as the framework, it has displayed excellent performance in chlorofluorocarbon and hydrofluorocarbon decomposition [10,11,12,13]. The catalytic behavior is related to its high specific surface area, strong acidity, and three-dimensional channel system. However, the amount of strong acidic sites and Lewis acidic sites are insufficient for CF_4_ decomposition.

Cerium (Ce) is a good promoter for catalyst, due to its high oxygen-storage capacity, high oxygen-vacancy concentration, and the facile Ce^4+^/Ce^3+^ redox cycle, which enhances the electron transfer that generates more acidic sites [14,15]. For example, de Rivas et al. [12] studied the 1,2-dichloroethane decomposition over the Ce/HZSM-5 catalyst, and they achieved a 90% conversion that was attributed to the addition of Ce which increased the Lewis acidic sites. Chen et al. [16] synthesized a [Ce-(1,3,5-benzenetricarboxylic acid)(H_2_O)_6_] catalyst, and achieved a good activity for toluene oxidation with conversion of T_90%_ at 223 °C, the authors concluded that the catalytic activity was due to the great amount of acidic sites on catalyst. Moreover, some research suggests that the sulfuric acid treatment is an excellent strategy to significantly increase the Lewis acidic sites on catalysts, as the sulfate ions act as Lewis acidic sites; meanwhile, they attracted electrons to create more new acidic sites [17,18]. Song et al. [19] modified the γ-Al_2_O_3_ with Ce and sulfuric acid treatment for CF_4_ catalytic decomposition, and found that the addition of Ce increased the acidic sites, the sulfuric acid treatment further enhanced the increasement, and the CF_4_ conversions were consistent with the amount of acidic sites. Although varied Lewis acid type catalysts can be applied in CF_4_ decomposition, a high temperature (over 700 °C) is required to decompose the CF_4_ molecule [20,21,22]. Therefore, we focused on developing a HZSM-5 based catalyst by modification with element Ce and sulfuric acid treatment for CF_4_ decomposition below 700 °C.

Given the above reasons, a series of modified HZSM-5 catalysts were developed to hydrolytic decompose CF_4_ at 500 °C. The aim of this work is to investigate the changes in the properties of the catalyst on the addition of Ce and acid treatment. The physicochemical properties of catalysts were characterized by different techniques. This study intended to elucidate the mechanisms of CF_4_ decomposition over HZSM-5 based catalysts.

## 2. Experimental Section

### 2.1. Catalyst Preparation

The Ce/HZSM-5 catalyst was prepared using the impregnation–calcination method from the commercial molecular sieve HZSM-5 (Si/Al = 18, mole ratio, Nankai Unv, Tianjin, China) calcined at 650 °C in N_2_ atmosphere for 5 h with an aqueous solution containing required amounts of cerium nitrate (Sinopharm Chemical Reagent Co., Ltd., Shanghai, China), followed by drying overnight at 80 °C. The Ce/HZSM-5 (10%Ce/HZSM-5) catalyst was crushed and sieved into 60–80 mesh. The Ce/HZSM-5 catalyst was impregnated with an aqueous solution containing H_2_SO_4_ (98%, Sinopharm Chemical Reagent Co., Ltd., Shanghai, China) under stirring for 24 h and dry for 12 h at 60 °C, the impregnating solution was adjusted to yield the same SO_4_^2−^ (12 wt%) as S/Ce/HZSM-5, finally, calcined at 650 °C for 5 h in N_2_ atmosphere. The S/Ce/HZSM-5 (12%S/10%Ce/HZSM-5) catalyst was crushed and sieved into 60–80 mesh.

### 2.2. Catalytic Activity Test

The hydrolytic decomposition of CF_4_ was carried out in a fixed-bed reactor. The temperature was maintained at 500 °C. In the aluminum production, the CF_4_ generated in electrolytic cell during the anode effect, the CF_4_ concentration in the flue gas is about 1%. Therefore, the gas flow consists of l% CF_4_, 35% H_2_O, and balanced by Ar. The water was pre-heated at 150 °C and then constantly introduced into the reaction system by using a syringe pump. The effluent gas was washed by aqueous potassium hydroxide to remove the produced HF, and then passed through a cold trap to remove water. Finally, the gas was analyzed by an online gas chromatography (GC-9790 II) equipped with a thermal conductivity detector (TCD).

### 2.3. Catalyst Characterization

The morphology of the samples was examined by scanning electron microscope (SEM, JSM-IT300LA, JEOL, Tokyo, Japan) with energy dispersive X-ray (EDX) analysis. The X-ray diffraction analysis was performed on a TTR III diffractometer (XRD, Rigaku, Tokyo, Japan). The chemical composition and state of the elements on catalyst surfaces were investigated by X-ray photoelectron spectroscopy (XPS, EscaLab 250Xi, Thermo Fisher, Waltham, MA, USA). The specific surface areas were measured with the N_2_ adsorption method on an ASAP analyzer (BET, ASAP2020, Micromeritics, Norcross, GA, USA). Ammonia temperature programmed desorption (NH_3_-TPD, AutoChem II 2920, Micromeritics, Norcross, GA, USA) analyses were performed using the following procedures. A 100 mg sample was pretreated at 550 °C, with helium flow of 30 mL/min for 1 h, and then cooled to 50 °C. Ammonia (10% NH_3_/He) was introduced to the catalyst for 1 h at 50 °C. After that, the sample was flushed with helium of 50 mL/min for 1 h to remove absorbed NH_3_, then the temperature was programmed to increase to 900 °C at a rate of 10 °C/min. The amount of ammonia desorbed from the catalyst was detected using TCD. Fourier transform infrared spectra (FT-IR, Nicolet iS50, Thermo Fisher, Waltham, MA, USA) of pyridine absorption were conducted using the following procedures. The sample was pressed and put into an IR cell, and then it was degassed at 400 °C under vacuum for 2 h to dehydrate. Next, the cell was cooled to room temperature, and the background signal was recorded. After that, pyridine vapor was introduced to the system until reaching adsorption equilibrium. The sample was evacuated out at 150 °C for 30 min followed by cooling down to 50 °C, and then spectral acquisition was performed.

## 3. Results and Discussion

### 3.1. Characterization of Catalysts

**Figure 1:**
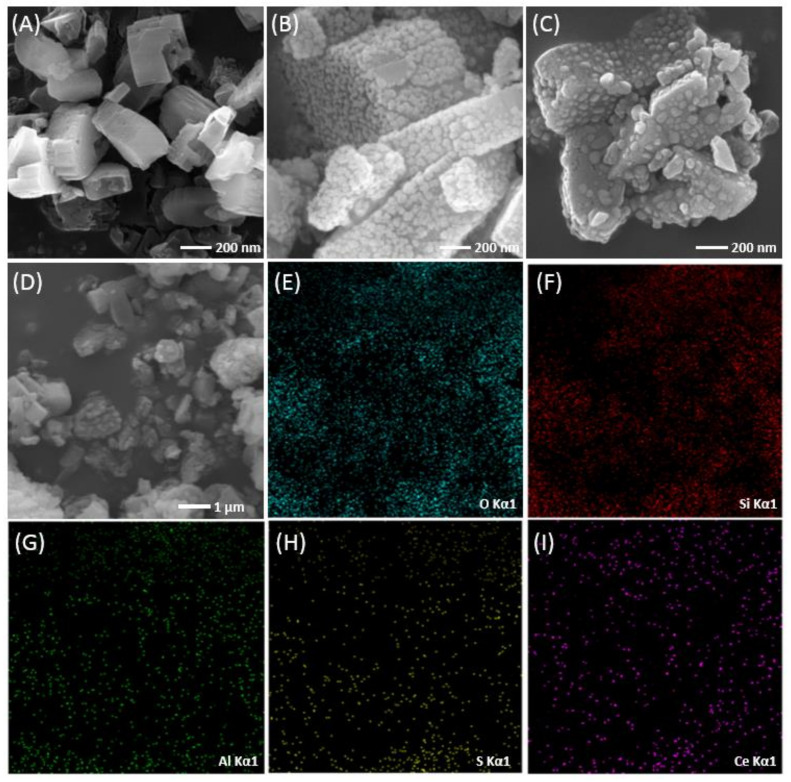
SEM of (**A**) HZSM-5, (**B**) Ce/HZSM-5, (**C**,**D**) S/Ce/HZSM-5, and SEM-EDS mapping for S/Ce/HZSM-5 (**E**–**I**).

The morphology and elemental distribution of catalysts were monitored by SEM and EDS mapping, as shown in Figure 1. The microscopic images of HZSM-5 (Figure 1A) exhibit the nano-blocky particles with a smooth surface. The modified catalyst Ce/HZSM-5 (Figure 1B) and the S/Ce/HZSM-5 (Figure 1C,D) exhibit rough surface, the catalysts were covered by small solid granule. The EDS mapping for S/Ce/HZSM-5 (Figure 1E–I) exhibits the uniform distribution of O, Si, Al, S and Ce, respectively.

**Figure 2:**
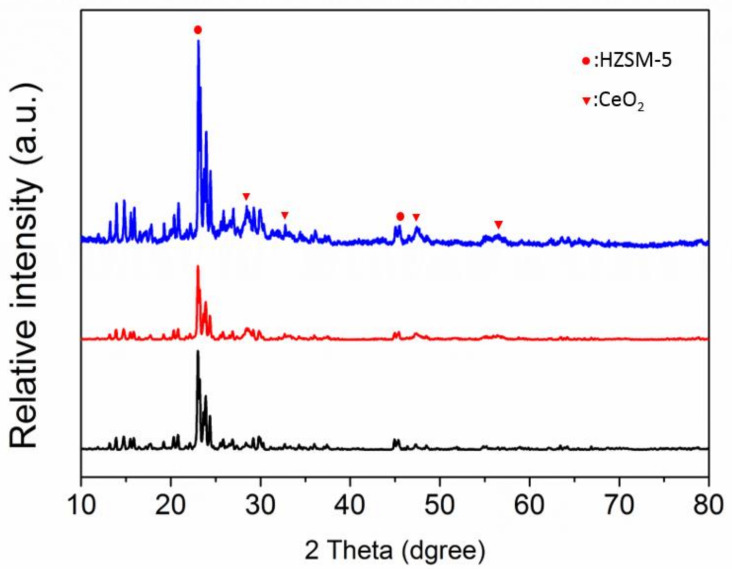
X-ray diffraction (XRD) patterns of the catalysts, (1) HZSM-5, (2) Ce/HZSM-5, and (3) S/Ce/HZSM-5.

The X-ray diffraction (XRD) was performed to investigate the crystal structure of the catalysts as in Figure 2. All the samples contain the HZSM-5 [23], and the peak of CeO_2_ (JCPDS:34-0394) is observed in modified samples, indicating the formation of CeO_2_ [24]. It should be noted that the peak of Ce_2_(SO_4_)_3_ is not observed in the S/Ce/HZSM-5 catalyst, which indicated the absence of reaction between Ce and sulfuric acid during the catalyst preparation step. The specific surface area and pore diameter of catalysts were analyzed, as listed in Table 1. The surface area of catalysts decreased from 248 m^2^ g^−1^ to 148 m^2^ g^−1^, and the pore diameter decreased from 2.38 nm to 2.28 nm after modified by Ce, these changes are in agreement with previous research [19,25], which may account for the formation of CeO_2_ on the surface of catalysts, in addition to element Ce, some cation ions were replaced by Ce, the CeO_2_ would block the original pore, results in the surface area and pore diameter decreasing. The surface area and pore diameter increased to 278 m^2^ g^−1^ and 2.28 nm, respectively, after the sulfate acid treatment. This may be due to the fact that acid treatment increased the amount of amorphous silica [26].

**Figure 3:**
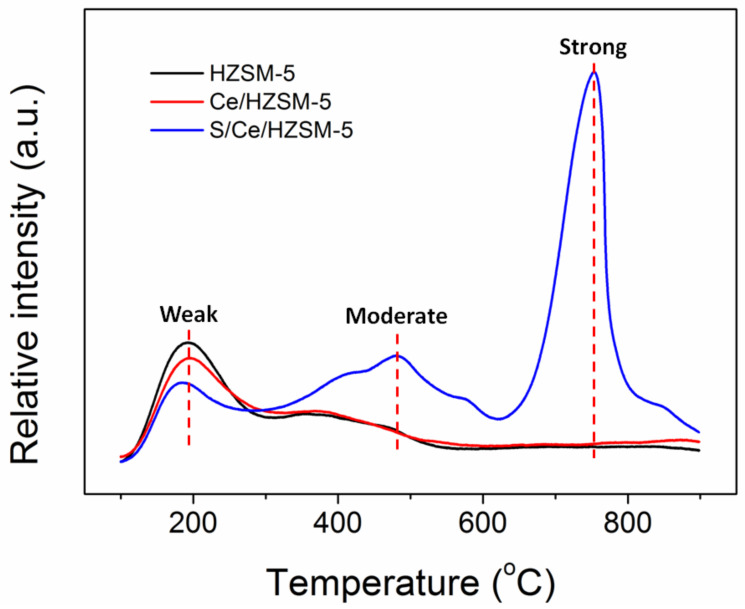
NH_3_-TPD profiles of catalysts.

The NH_3_-TPD experiments were conducted to analyze the different kinds of acidic sites in the catalysts. There are three kinds of acidic site according to the desorption temperature of NH_3_ on catalysts: weak acidic sites (T < 250 °C), moderate acidic sites (250 °C < T < 600 °C), and strong acidic sites (T > 600 °C) [27,28,29]. The TPD results are shown in Figure 3 and Table 2, the HZSM-5 catalyst exhibited the most weak acidic sites (663 µmol g^−1^) and the second highest amount of moderate acidic sites (267 µmol g^−1^), the strong acidic site was not observed. The addition of Ce decreased the amount of weak acidic sites to 290 µmol g^−1^ and increased the moderate acidic sites to 544 µmol g^−1^, but still, no strong acidic site was observed. After further acid treatment, the amount of weak acidic sites decreased to 273 µmol g^−1^ and the amount of moderate acidic sites increased to 855 µmol g^−1^, the amount of strong acidic sites dramatically increased to 1274 µmol g^−1^, which dominated over others. These findings strongly indicated that the impregnation with sulfate group influenced the acidic properties of catalysts, the amount of moderate and strong acidic sites significantly increased; moreover, the ratio of (strong + moderate)/weak was in the following order: S/Ce/HZSM-5(7.80) > Ce/HZSM-5(1.88) > HZSM-5(0.40).

**Figure 4:**
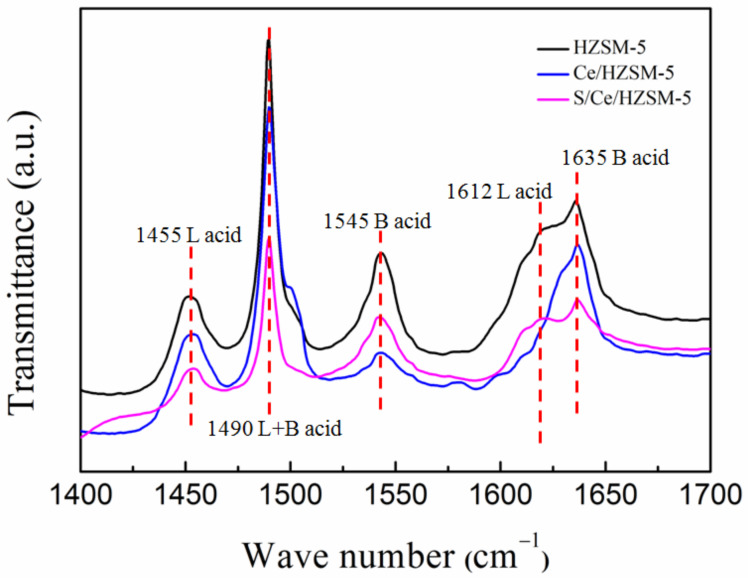
FT-IR spectra of pyridine adsorption on catalysts.

In order to gain more information on acidic properties of catalysts, the FT-IR spectra of pyridine adsorption experiments are conducted as shown in Figure 4 and Table 3. The spectra are recorded over a frequency range from 1400 cm^−1^ to 1700 cm^−1^, where characteristic vibration modes of adsorbed pyridine will appear. According to the references [30,31], the characteristic wave number of 1455 cm^−1^, 1612 cm^−1^ could be assigned to the pyridine species coordinatively adsorbed on Lewis acid sites, while the wave number of 1545 cm^−1^, 1635 cm^−1^ assigned to pyridinium ion formed on Brønsted acid sites, the 1490 peak was attributable to the L+B acid sites. The amounts of L-acid site on HZSM-5, Ce/HZSM-5 and S/Ce/HZSM-5 were 120.57 µmol g^−1^, 138.26 µmol g^−1^ and 156.84 µmol g^−1^, respectively. The amount of Brønsted acid sites showed the reversed trend, they were 233.91 µmol g^−1^, 83.61 µmol g^−1^ and 69.97 µmol g^−1^, respectively. The ratio of Lewis/Brønsted was in the following order: S/Ce/HZSM-5(2.24) > Ce/HZSM-5(1.65) > HZSM-5(0.52). The results indicated that the addition of Ce increased the amount of Lewis acidic sites while decreasing the Brønsted acidic sites; furthermore, the acid treatment enhanced the influence.

### 3.2. Catalytic Performance of CF_4_ Decomposition

**Figure 5:**
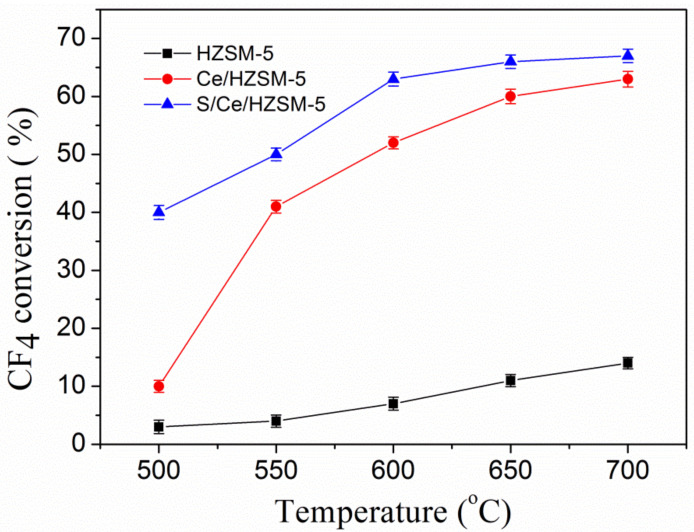
CF_4_ conversion reaction over catalysts at different temperatures.

The conversion reactions of CF_4_ over catalysts were conducted at different temperature, as shown in Figure 5. The HZSM-5 exhibited no catalytic activity below 600 °C, and only 14% conversion of CF_4_ while the temperature increased up to 700 °C. The Ce/HZSM-5 exhibited a higher CF_4_ conversion compared to HZSM-5, the CF_4_ conversions at 500 °C, 550 °C, 600 °C, 650 °C and 700 °C were 10%, 41%, 52%, 60% and 63%, respectively. As for the S/Ce/HZSM-5 catalyst, the CF_4_ conversion was further enhanced, they were 41%, 50%, 63%, 66% and 67%, respectively. Moreover, at the temperature of 500 °C, the conversion of S/Ce/HZSM-5 was four times higher than that of Ce/HZSM-5. These results indicated that the acid treatment significantly increased the catalytic activity of Ce/HZSM-5, which showed a good agreement with the results of BET, NH_3_-TPD and FT-IR.

**Figure 6:**
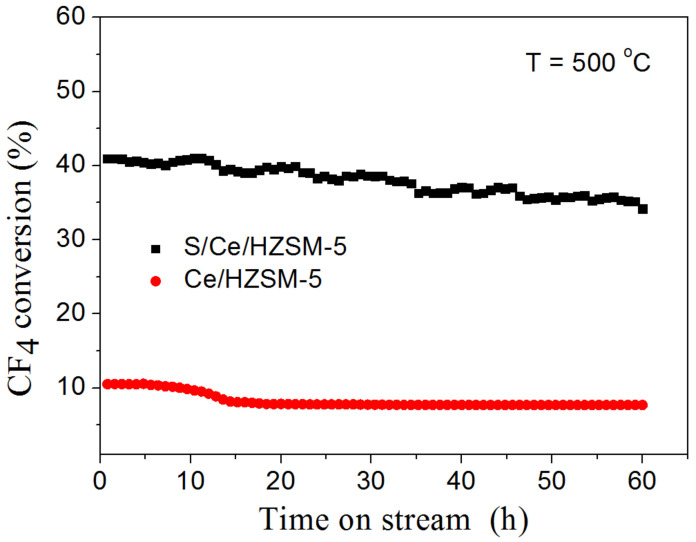
CF_4_ conversion over catalysts with time on stream.

The stability of catalysts was tested for 60 h at 500 °C as shown in Figure 6. The CF_4_ conversion over S/Ce/HZSM-5 decreased from 41% to 34% (17% loss), while the conversion over Ce/HZSM-5 decreased from 11% to 7% (27% loss). The catalytic stability of S/Ce/HZSM-5 was much higher than that of Ce/HZSM-5. This may be due to the increment of acidic sites by acid treatment.

### 3.3. Mechanism Analysis of Hydrolytic Decomposition of CF_4_

**Figure 7:**
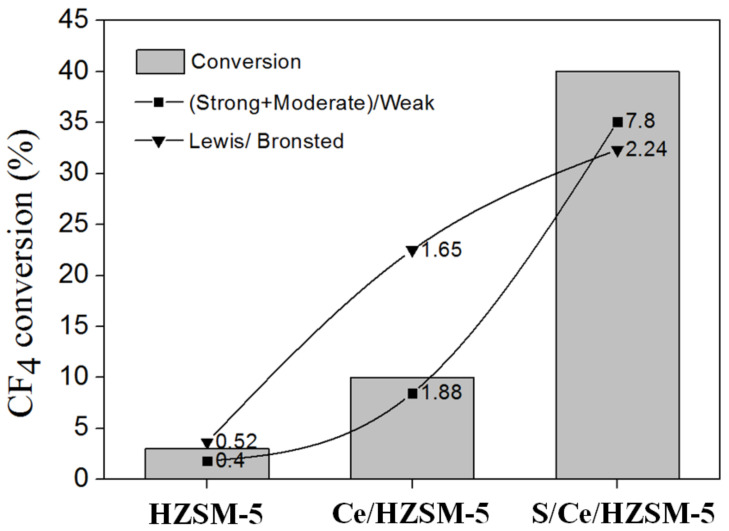
Correlation between CF_4_ conversion and ratio of (strong + moderate)/weak and Lewis/Brønsted.

In order to demonstrate the main factor that promoted the catalytic activity of catalysts, the close correlation between CF_4_ conversion and the ratio of (strong + moderate)/weak and Lewis/Brønsted were performed as shown in Figure 7. The correlation showed that the conversion is related to the ratio of (strong + moderate)/weak and the ratio of Lewis/Brønsted. Therefore, we concluded that the strong + moderate acidic sites and the Lewis acidic sites, are the main factor that promoted the activity of the catalysts. The HZSM-5 exhibited no catalytic activity due to its low ratio of (strong + moderate)/weak and Lewis/Brønsted. Similarly, it has been reported that the Lewis acid sites and strong + moderate acid sites played a promoting role in the decomposition of hydrofluorocarbons [32,33,34]. The results strongly suggest that the direct participation of the acid sites in CF_4_ decomposition is probably a step for subtracting fluorine atom from CF_4_ molecule by Lewis acid sites. The addition of element Ce and the acid treatment could significantly influence the acidic property, resulting in increasing the catalytic activity and stability of catalysts in CF_4_ decomposition.

**Figure 8:**
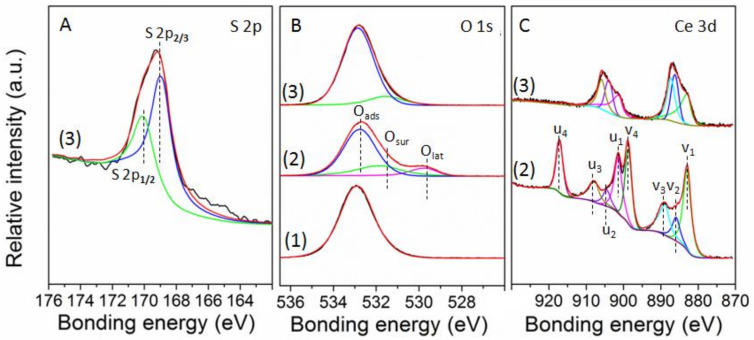
X-ray photo electron spectroscopy (XPS) spectra of catalyst, (**A**): S 2p for catalyst, (**B**): O 1s for catalysts, and (**C**): Ce 3d for catalysts. (1) HZSM-5, (2) Ce/HZSM-5, and (3) S/Ce/HZSM-5.

In order to acquire insights into the surface chemical composition of catalysts, X-ray photoelectron spectroscopy (XPS) analysis of catalysts was carried out and shown in Figure 8 and Table 4. A curve-fitting for this analysis was carried out after Shirley-type background subtraction using a combination of Gaussian and Lorentzian functions. Figure 8A presented the S 2p core level spectra, the two major binding energy values were located at 168.90 eV and 170.10 eV, attributing to S 2p_1/2_ and S 2p_2/3_ respectively, which were ascribed to S^6+^ from SO_4_^2−^. According to references [17,18,35], the S^6+^ from SO_4_^2−^ could significantly increase the amount of Lewis acidic sites, which showed a good agreement with the results of NH_3_-TPD and FT-IR. The O 1s core level spectra of catalysts were shown in Figure 8B, there are three major binding energy values located at range 528.9 to 529.5 eV, range 530.0 to 531.6 eV, and range 531.9 to 533.2 eV, attributing to O_lat_, O_sur_ and O_ads_, respectively [36,37]. After the acid treatment, the peak of O_lat_ disappeared, the intensity of peak for O_ads_ increased, it could be concluded that the O_lat_ were transformed to O_ads_, meanwhile, some of the O_lat_ bonded with S^6+^ to form SO_4_^2−^, then increased the amount of Lewis acidic sites. The Ce 3d core level spectra of catalysts are shown in Figure 8C, the peak v_2_ (885.9 eV–886.3 eV) and peak u_2_ (904.0 eV–904.5 eV) were ascribed to Ce^3+^, the peak v_1_ (883.0 eV), peak v_3_ (887.3 eV–889.1 eV), peak v_4_ (898.8 eV), peak u_1_ (901.5 eV), peak u_3_ (905.9 eV–907.7 eV) and peak u_4_ (917.3 eV) were ascribed to Ce^4+^ [38,39], after the sulfuric acid treatment, the peak u_4_ and peak v_4_ disappeared, the Ce^3+^/Ce increased from 25.9% to 31.0%, indicating that the sulfuric acid treatment reduced the Ce^4+^ to Ce^3+^ because they attracted electrons to create more Lewis acidic sites, which showed a good agreement with the CF_4_ conversion performance and the results of NH_3_-TPD and FT-IR.

## 4. Conclusions

We modified the commercial HZSM-5 with the addition of Ce(Ce/HZSM-5) and further sulfuric acid treatment (S/Ce/HZSM-5). The S/Ce/HZSM-5 catalyst achieved a 41% CF_4_ conversion at 500 °C which is four times higher than that over Ce/HZSM-5, while the HZSM-5 exhibited no catalytic activity. The close correlation between CF_4_ catalytic conversion and acidic property indicated that the Lewis acidic sites and moderate + strong acidic sites are the main factors that promoted the activity of the catalysts. The effects of modification were confirmed by using NH_3_-TPD, FT-IR of pyridine adsorption and XPS methods. These results indicate that the acidity properties of catalysts are strongly influenced by the addition of Ce and further sulfuric acid treatment, and then significantly improved the catalytic activity in the CF_4_ decomposition process, which is an acid-catalyzed reaction.

## Figures and Tables

**Table 1:** Specific surface area, pore diameter for the catalysts.

Catalysts	BET Surface Area (m^2^ g^−1^)	Pore Diameter (nm)
HZSM-5	248	2.38
Ce/HZSM-5	148	2.28
S/Ce/HZSM-5	278	2.63

**Table 2:** NH_3_-TPD acidic sites results for the catalyst.

Catalysts	Amount of Acid Site (µmol g^−1^)				
	Weak	Moderate	Strong	Total	(Strong + Moderate)/Weak
HZSM-5	663	267	0	930	0.40
Ce/HZSM-5	290	544	0	834	1.88
S/Ce/HZSM-5	273	855	1274	2402	7.80

**Table 3:** FT-IR spectra of pyridine adsorption results for the catalyst.

Catalysts	Amount of Acid Site (µmol g^−1^)		
	L-Acid Site	B-Acid Site	Lewis/Brønsted (L/B)
HZSM-5	120.57	233.91	0.52
Ce/HZSM-5	138.26	83.61	1.65
S/Ce/HZSM-5	156.84	69.97	2.24

**Table 4:** Surface atomic distribution of element O and Ce.

Catalysts	Distribution (%)			
	O_lat_	O_sur_	O_ads_	Ce^3+^/Ce
HZSM-5	0	0	100	/
Ce/HZSM-5	10.5	22.0	67.5	25.9
S/Ce/HZSM-5	0	10.1	89.9	31.0

## Data Availability

The data supporting this study are available from the corresponding author upon reasonable request.
